# Improving Early Discharges With an Electronic Health Record Discharge Optimization Tool

**DOI:** 10.1097/pq9.0000000000000301

**Published:** 2020-05-18

**Authors:** Michael F. Perry, Charlie Macias, Juan D. Chaparro, Allison C. Heacock, Kenneth Jackson, Ryan S. Bode

**Affiliations:** From the * Division of Pediatric Hospital Medicine, Nationwide Children’s Hospital, Columbus, Ohio; † Quality Improvement Service; Nationwide Children’s Hospital, Columbus, Ohio; ‡ Division of Clinical Informatics, Nationwide Children’s Hospital, Columbus, Ohio; § Division of Internal Medicine and Pediatrics, Nationwide Children’s Hospital, Columbus, Ohio; ¶ Biostatistics Resource, Nationwide Children’s Hospital, Columbus, Ohio; ∥ Center for Biostatistics, The Ohio State University, Columbus, Ohio.

## Abstract

**Introduction:**

Delays in hospital discharge can negatively impact patient care, bed availability, and patient satisfaction. There are limited studies examining how the electronic health record (EHR) can be used to improve discharge timeliness. This study aimed to implement an EHR discharge optimization tool (DOT) successfully and achieve a discharge before noon (DBN) percentage of 25%.

**Methods:**

We conducted a single-center quality improvement study of patients discharged from 3 pediatric hospital medicine teaching service teams at a quaternary care academic children’s hospital. The multidisciplinary team created a DOT centrally embedded within the care team standard workflow to communicate anticipated time until discharge. The primary outcome was the monthly percentage of patients discharged before noon. Secondary outcomes included provider utilization of the DOT, tool accuracy, and patient length of stay. Balancing measures were 7- and 30-day readmission rates.

**Results:**

The DBN percentage increased from 16.4% to an average of 19.3% over the 13-month intervention period (*P* = 0.0005). DOT utilization was measured at 87.2%, and the overall accuracy of predicting time until discharge was 75.6% (*P <* 0.0001). Median length of stay declined from 1.75 to 1.68 days (*P* = 0.0033), and there was no negative impact on 7- or 30-day readmission rates.

**Conclusion:**

This initiative demonstrated that a highly utilized and accurate discharge tool could be created in the EHR to assist medical care teams with improving DBN percentage on busy, academic teaching services.

## INTRODUCTION

Hospitals have increasingly focused on optimizing patient flow to improve care and reduce potential harm.^1^ Discharge delays prevent newly admitted patients from being placed in the right bed at the right time, often leading to emergency department overcrowding, patient dissatisfaction, and adverse events.^2–4^ During high-census periods, the lack of available inpatient beds can lead to the diversion of critically ill patients, canceled surgical procedures, or postponement of scheduled admissions. A recent survey of academic centers revealed that many had adopted initiatives to improve discharge time to mitigate these problems.^5^

Several published improvement efforts have shown an increase in the percentage of early discharges.^6–10^ These studies have included identification of early discharges, checklists, team huddles, separate physician-care manager rounding, and physician incentives. Wertheimer et al^8^ outlined a series of interventions improving the discharge before noon (DBN) percentage on 2 medical units from 11% to 38%, whereas Molla et al^9^ demonstrated a 7.5% point increase in DBN using Lean Six Sigma methodology.

Tyler et al^11^ implemented a discharge readiness report within the electronic health record (EHR) and hypothesized its potential positive impact on the discharge process. This study raised questions about the ability to leverage the EHR to improve discharge efficiency. With EHR systems becoming near-universal in the United States, interventions within the EHR may provide easily transferable opportunities to improve discharge.^12^

The study objective was to create a discharge optimization tool (DOT) within the EHR to improve early discharges in 3 pediatric hospital medicine teaching services. We hypothesized that visual cues displaying a patient’s anticipated time until discharge would help medical team members prioritize workflow toward patients nearing discharge. We aimed to increase the percentage of discharges before noon (DBN) from a baseline of 16.4% in the 13 months before February 2018 to 25% by April 2019.

## METHODS

### Setting

Nationwide Children’s Hospital in Columbus, Ohio, is a 527-bed freestanding pediatric academic center with over 18,000 admissions and 90,000 emergency department visits each year. Our study included both inpatient and observation status patients discharged from the 3 pediatric hospital medicine teaching teams. Each team consisted of an attending hospitalist physician, pediatric residents, medical students, physician assistant students, and resident assistants, who are administrative assistants assigned to inpatient teaching services. We excluded patients whose discharge diagnosis was for a medically stable behavioral health concern (eg, suicidal ideation, self-harm by intentional ingestion) since their discharge was dependent on placement by the psychiatry service. All obtained admission and discharge data came from our enterprise data warehouse. This project was deemed quality improvement research by the Nationwide Children’s Hospital Institutional Review Board, so it did not require formal review per policy.

### Improvement Team

Formed in 2017, our multidisciplinary team included pediatric hospitalist physicians, clinical informatics specialists, quality improvement service line coordinator, nursing manager, registered nurses, chief resident, and resident assistants. Based on the Institute for Healthcare Improvement Model for Improvement methodology,^13^ the team developed a key driver diagram to formulate our aim statement, identify barriers, and implement our intervention. Our team met monthly to review the DBN and DOT data and visualized results on a statistical process control p-chart.

### Intervention

**Fig. 1.**
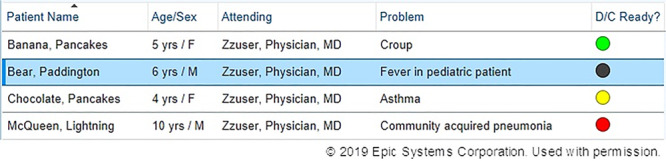
Sample screenshot of a patient list with the discharge optimization tool. Reprinted with permission from Epic Systems Corporation. F indicates female; M, male; D/C, discharge.

Our multidisciplinary team worked with clinical informatics specialists to develop the DOT for provider teams to communicate anticipated time until discharge for each patient. The tool was a dedicated column incorporated into the medical team’s patient list within the EHR, Epic (Epic Systems Corporation, Verona, Wisc.) (Fig. 1). Given the longitudinal and dynamic nature of the discharge process, we created a color-coded “dot” system with 4 designations as to the anticipated time until discharge: (1) green—discharge in <12 hours; (2) yellow—discharge in 12–24 hours; (3) red—discharge in 24–48 hours; and (4) black—discharge not anticipated or undetermined at this time. The tool provided an easily visible notification regarding a patient’s discharge readiness status, for example, patients designated with green dots should be discharged sooner than patients with red dots.

The integration of the DOT into the EHR occurred in February 2018. Education to on-service hospitalist physicians, residents, students, and resident assistants occurred via email and in-person demonstrations. Medical teams were asked to discuss a patient’s anticipated time until discharge and select the appropriate color-coded discharge readiness status. Teams were expected to update the DOT upon admission, then at least twice a day for the remainder of the patient's admission, once after family-centered rounds and before 5:30 pm resident sign out.

### Measures

#### Primary Outcome: DBN

Our primary outcome of interest was the DBN percentage occurring in pediatric hospital medicine teaching services. We obtained discharge data during the baseline period (January 2017 to January 2018) and the postintervention period (March 2018 to March 2019). We excluded February 2018 from analysis to allow for education and implementation of the DOT. The team defined DBN as any inpatient discharge occurring between 12:01 am and noon. We calculated monthly DBN percentages by dividing the total number of DBN each month by the total number of patients discharged during the month. Before discharge, patients were required to have printed discharge instructions called the After Visit Summary (AVS). We used AVS printed by 11:00 am as a leading process measure.

#### Secondary Outcomes: Tool Utilization, Selection Accuracy, and Length of Stay

We followed 2 secondary measures related to the discharge tool: (1) tool utilization, defined as a patient with at least one discharge readiness assessment made from the time of admission to the time of discharge and (2) selection accuracy, defined as the patient’s last readiness assessment selection correctly matching the time it took for their discharge. We obtained data for our DOT through reports generated from our enterprise data warehouse. Also, we tracked the length of stay (LOS) hypothesizing that improved DBN percentage should decrease the overall length of hospitalization.

#### Balancing Measures: Readmission Rates

Our balancing measures were all-cause 7- and 30-day readmission rates, which we obtained using the hospital’s regularly reported data from the data warehouse center with the exclusion of behavioral health patients.

### Analysis

We displayed our primary outcome measure of DBN and secondary outcome measures of DOT utilization and accuracy on statistical process control p-charts. We used Nelson rules to define special versus common cause variation.^14^ The data were subset from Nationwide Children’s EHR, exported into an Excel spreadsheet (Microsoft, Redmond, Wash.), and imported back into a SAS dataset (SAS, Cary, N.C.). Univariate comparisons of outcomes for the process metric of AVS printed before 11:00 am and DBN were conducted via Student 2-sample *t* tests (assuming equal variances). Effect sizes were estimated via odds ratios and corresponding 95% confidence intervals. LOS was analyzed via nonparametric Wilcoxon rank-sum tests, given the strong deviation from a normal distribution. Chi-square tests were used to quantify the relationship between the last assessment value selected and the actual time from the last assessment to discharge and to examine all-cause readmission rates.

## RESULTS

### Primary Outcome: DBN

**Fig. 2.**
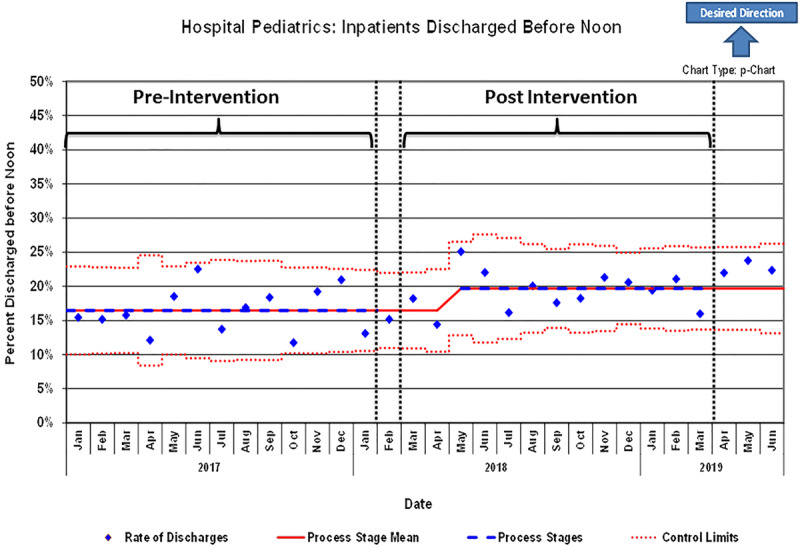
Statistical process control p-chart for patients discharged before noon pre- and postimplementation of the discharge optimization tool.

During the 13-month baseline period, we identified 4,033 discharges from the pediatric hospital medicine teaching services. After our 1-month implementation period in February 2018, we identified 4,849 discharges in the 13-month postintervention period. The average monthly DBN percentage improved from 16.4% to 19.3%, representing an 18% relative increase in our DBN rate (*P* = 0.0005; odds ratio, 1.213; 95% confidence interval, 1.087–1.353). The control chart showed a desired upward centerline shift starting in May 2018 and sustained for the duration of the project (Fig. 2). The process measure, AVS printed before 11 am percentage, also improved from 13.6% to 15.8% (*P* = 0.0059; odds ratio, 1.179; 95% confidence interval, 1.048–1.326).

### Secondary Outcomes: DOT Utilization and Accuracy and LOS

**Fig. 3.**
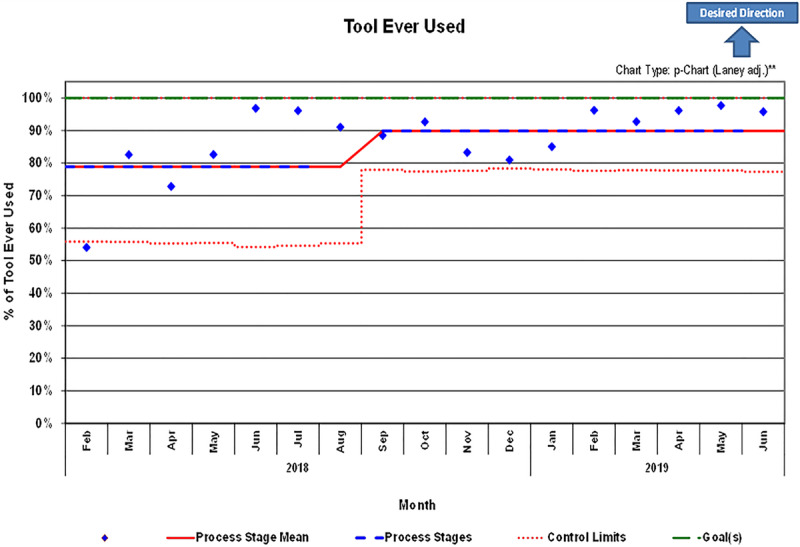
Statistical process control p-chart for utilization of discharge optimization tool at least once during patient hospitalization. **Alternative control limit calculations have been used to compensate for overdispersion (more variation than predicted) in the data of one or more process stages.

**Fig. 4.**
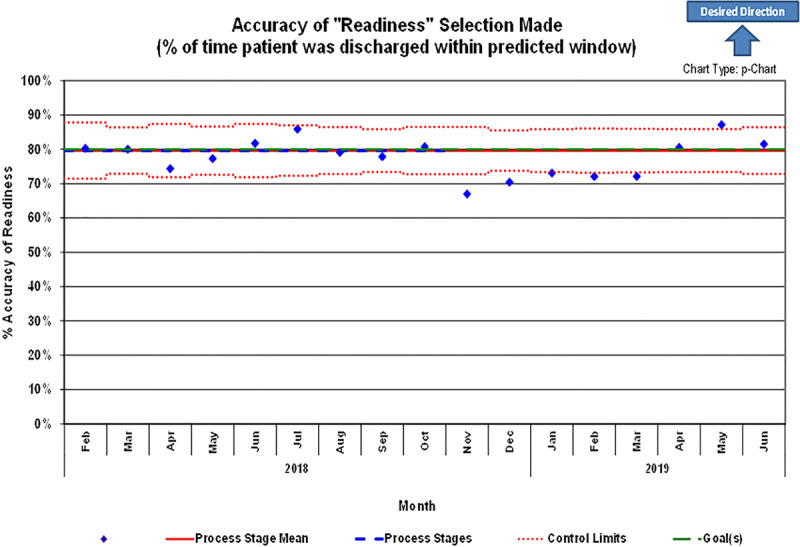
Statistical process control p-chart for accuracy of last readiness assessment correctly predicting a patient’s actual time until discharge.

Of the 4,849 discharges in the postintervention period, there were 4229 encounters (87.2%), where the DOT was used at least once during the hospitalization (Fig. 3). In contrast, 2,953 encounters (69.8%) had the DOT used more than once. Overall tool accuracy measured at 75.6% (*P <* 0.0001). A control p-chart displayed monthly percentages of tool accuracy (Fig. 4). We observed a statistically significant difference (*P* = 0.0033) between the baseline LOS distribution (median, 1.75 days) and the postintervention LOS distribution (median, 1.68 days).

### Balancing Measures: Readmission Rates

The DOT did not impact either 7- or 30-day readmission rates. The 7-day all-cause readmission rate (3.0% versus 2.4%; *P* = 0.0888) and 30-day (6.3% versus 5.9%; *P =* 0.4820) all-cause readmission rates improved postimplementation but did not reach statistical significance.

## DISCUSSION

Discharge efficiency remains a top priority for hospitals despite numerous identified barriers.^5,15–17^ As health care systems increasingly rely on EHRs, there are new and innovative ways within standard EHR workflow to improve discharge efficiency. In our study of nearly 9,000 discharges, we successfully demonstrated how a DOT integrated into the EHR could increase the number of discharges before noon. We were able to increase the DBN percentage of 3 pediatric hospital medicine teaching services from 16.4% to 19.3% without having adverse effects on readmission rates. More importantly, we created a visual tool that providers used with notable accuracy, correctly predicting time until discharge 76% of the time.

Previous studies utilizing the EHR to improve patient flow and efficiency primarily focused on the creation of electronic dashboards (e-dashboards).^18–20^ While e-dashboards have some noteworthy benefits, they often only provide high-level overviews and are not easily replicated in other institutions. Furthermore, they typically exist within a domain of the EHR not easily or frequently accessed by all members of a health care team.

Our novel DOT provided easy-to-understand, color-coded visual cues regarding a patient’s anticipated time until discharge and was centrally embedded, allowing for easy accessibility and visualization. Although quality improvement initiatives may change providers’ behaviors due to increased attention from being observed (ie, the Hawthorne effect), previous efforts to improve discharge efficiency at our institution were not successful. We postulate that our DOT intervention led to positive change with visual cues, which helped teams prioritize discharge tasks to first focus on patients more likely to be discharged home sooner. Also, we suspect that the tool promoted improved communication among care team members regarding a patient’s discharge status. Given its intuitive and user-friendly concept, the DOT required minimal training, which allowed for quick and sustained adoption. Also, the relatively easy technical nature of this EHR implementation enables it to be generalizable to both community and academic hospitals as well as other EHR vendors. These numerous benefits of the DOT allowed us to achieve a statistically significant improvement in the DBN percentage without adversely affecting readmission rates.

This initiative improved discharge efficiency on pediatric hospital medicine inpatient services historically known for high patient turnover, the short LOSs, and high census during winter months. Although we observed a modest percentage point increase in DBN, a small absolute increase spread across thousands of discharges can have a substantial impact on a health care system. DBN has become a commonly used institutional metric, although some have argued against its value. Critics have speculated that patients may remain admitted longer than necessary to achieve DBN the following day.^21^ Our institutional culture, particularly in pediatric hospital medicine, is to discharge when the patient is medically ready. The statistically significant difference in the baseline versus postintervention LOS underscores this culture and reaffirms the results of our study. This improvement in LOS mirrors previous discharge efficiency studies that also demonstrated a reduction in LOS.^8,22,23^

This study had several limitations. We did not study variables that may prolong discharge, such as transportation availability, parental preference, or medical comorbidities.

Second, we had no LOS exclusion criteria, so patients with relatively short LOS, such as same-day admit/discharge, were included and may have contributed to not reaching our goal DBN rate of 25%. Pediatric hospital medicine patients generally have shorter lengths of stay, so this patient population may not generalize to other subspecialty services. Third, as a well-resourced academic center, we have a robust health information technology infrastructure that contributed to our project. Also, the numerous care team members associated with academic teaching services provided more opportunities to use the DOT and complete discharge-related tasks. This structure may not be available at all institutions, although ease of development and implementation makes the DOT generalizable to other academic centers.

We were unable to reach our goal DBN percentage, but our group has continued to monitor DBN rates regularly and explore new interventions. We plan to create standard workflow expectations associated with each color code, such as ensuring a patient has discharge transportation when given a yellow designation status. Other proposed interventions include spread to other pediatric subspecialty services, more direct communication with the family regarding anticipated discharge date, and development of diagnosis-specific clinical pathways related to discharge readiness and efficiency.

## CONCLUSIONS

We successfully developed a DOT embedded within the EHR, which improved our discharge timeliness. Our success with the DOT suggests that visual cues centrally embedded in the standard workflow may enhance team communication and task prioritization, ultimately resulting in improved discharge efficiency.

## DISCLOSURE

The authors have no financial interest to declare in relation to the content of this article.
